# Improving Mechanical Coffee Drying with Recycled Insulating Materials: A Thermal Efficiency and Economic Feasibility Analysis

**DOI:** 10.3390/foods15020367

**Published:** 2026-01-20

**Authors:** Valentina Cruz-Ospina, Eduardo Duque-Dussán, Juan R. Sanz-Uribe

**Affiliations:** Postharvest Discipline, National Coffee Research Center—Cenicafé, Km 4. Antígua Vía Chinchiná-Manizales, Manizales 170009, Colombia; valentina.cruz@cafedecolombia.com (V.C.-O.); juanr.sanz@cafedecolombia.com (J.R.S.-U.)

**Keywords:** circular economy, *Coffea arabica* L., energy losses, expanded polystyrene, forced convection, heat transfer, mass transfer

## Abstract

Mechanical coffee drying is an energy-intensive stage of postharvest processing that directly affects product quality and production costs. This study evaluated the technical and economic feasibility of using expanded polystyrene (EPS) as a thermal insulation material to improve the performance of a mechanical coffee dryer and to demonstrate its potential for sustainable reuse. Experiments were conducted using a total of 210 kg of wet parchment coffee (*Coffea arabica* L. var. Cenicafé 1) per treatment, corresponding to three experimental replicates of 70 kg each, dried at 50 ± 2 °C, comparing an EPS-insulated dryer (0.02 m thickness) with a non-insulated control. A theoretical model based on steady-state heat transfer through series resistances estimated energy losses and system efficiency for different insulating materials. Theoretical results indicated that EPS, polyethylene foam, and cork reduced heat losses by 58.1%, 54.3%, and 50.9%, respectively. Experimentally, EPS reduced drying time by 7.82%, fuel consumption by 13.9%, and energy demand by 9.5%, while increasing overall efficiency by 6.7% and reducing wall heat losses by 37.7%. Improved temperature stability enhanced heat retention and moisture migration behavior. Economically, EPS reduced operating costs, yielding annual savings of USD 81.5, a 0.45-year payback period, and an annual return on investment (ROI) of 10.86, confirming its viability as a cost-effective and sustainable solution for improving energy efficiency in mechanical coffee drying.

## 1. Introduction

Coffee is one of the agricultural products with the highest volume of trade worldwide [1,2]. Post-harvest processing methods vary according to the agroecological and cultural conditions of each producing region. The transformation of ripe fruits into dry coffee involves a sequence of operations whose technical characteristics depend on the processing type (wet, semi-dry, or dry) [3,4]. Drying is a common and critical stage across all methods, aimed at reducing grain moisture to the hygroscopic equilibrium point to prevent microbial growth and preserve quality [5]. Although coffee is not botanically a cereal grain, it is industrially treated as one, as it is a seed that must be dried before storage and commercialization [6]. Therefore, coffee exhibits the highest drying energy requirements among the grains, particularly for whole fruits (naturals), where moisture decreases from about 75% to 10–12% on a wet basis (wb) [7,8]. Parchment coffee presents similar challenges, not only because moisture must be reduced from 53% to 10–12% (wb) but due to the formation of an internal air chamber between the endocarp and endosperm due to differential shrinkage during drying [9].

The drying process involves the migration of moisture from the seed’s interior to its surface, followed by its exchange with the surrounding air. This exchange is driven by a vapor pressure gradient between liquid and gaseous phases [10], which increases at the grain–air interface and intensifies as drying air temperature rises through solar or mechanical heating [11]. Both drying methods differ in their physical principles, energy efficiency, operating times and adaptability to climatic conditions [12,13]. Solar drying uses incident radiation as a thermal source to increase the temperature inside the drying chamber [14], aided by airflow from natural convection or wind. In Colombian coffee regions, traditional solar drying remains common due to its accessibility, but it is limited by poor temperature control, long processing time, and non-uniformity in drying [15].

Mechanical drying allows precise control of temperature and airflow, maintaining uniform conditions and achieving homogeneous products [13,16]. Unlike solar drying, which pauses at night, mechanical dryers operate continuously regardless of environmental factors, reducing processing times and ensuring consistent drying quality [17,18,19]. These advantages have encouraged their growing adoption in Colombia, where high production volumes and harvest periods that often coincide with rainy seasons limit the effectiveness of solar drying [20,21]. However, most commercial dryers, typically made of steel with capacities around 250 kg, lack insulation to minimize manufacturing costs, leading to high thermal energy demand [22,23,24]. Consequently, there is increasing interest in alternative insulating materials that are effective, affordable, and easy to install.

In the pursuit of greater energy efficiency, the incorporation of insulating materials has proven to be a key strategy for optimizing thermal performance in various systems [25]. Among these materials, EPS stands out for its effectiveness in maintaining stable internal conditions in chambers or coated structures [26]. This is mainly due to its low thermal conductivity, typically ranging between 0.032 and 0.038 W m^−1^ K^−1^ [25,26], which has contributed to improving overall thermal efficiency in industrial systems, shortening operating times, and reducing specific energy demand by up to 70% [27]. However, once it reaches the end of its service life, there are limited options for recycling or reusing it, mainly because of its low density and contamination with other materials [28]. As a result, large quantities of EPS are often improperly discarded, contributing to long-term environmental pollution [29].

Considering its limited recyclability and environmental persistence, identifying sustainable reuse pathways—such as employing recycled EPS as an insulating material—could provide both energy and environmental benefits [30]. Its low cost, light weight, high thermal resistance, and wide availability make it an attractive candidate for improving energy efficiency in mechanical coffee drying systems [31,32]. Nevertheless, experimental validation is still required to quantify its impact on heat conservation, thermal stability, and fuel consumption, particularly in cases where biofuels such as coffee husk are used.

Based on this background, the present study aimed to evaluate the effect of the use of expanded polystyrene in a mechanical coffee drying system on the energy efficiency of the process. The research was developed in the following stages: In the first phase, an experimental evaluation was carried out considering variables such as the total drying time, the external surface temperature, the distribution of temperature and humidity inside the dryer, the drying dynamics of the grain and the specific demand of energy and fuel consumption. Subsequently, a theoretical analysis of heat transfer was made, considering the energy losses due to conduction and convection, the estimated fuel consumption and the overall thermal efficiency of the system using different insulating materials. Additionally, the economic impact of the implementation of insulation was estimated using indicators such as the unit cost per kilogram of dry coffee, the return on investment (ROI) and the net savings in fuel.

## 2. Materials and Methods

### 2.1. Experimental Evaluation

#### 2.1.1. Study Site and Material Collection

The evaluation was carried out at the facilities of the Postharvest discipline at the National Coffee Research Center of Colombia—Cenicafé (Manizales, Caldas, Colombia; 4.991889° N, 75.597139° W; 1306 m). During the months of July and August 2025, average temperatures are 22 ± 4 °C and the ambient relative humidity is 78 ± 2.5% [33,34]. *Coffea arabica* L. var. Cenicafé 1 processed using the wet method was used as the product to dry. For each treatment, 210 kg of wet parchment coffee were used, divided into three replicates (70 kg per repetition), with an initial moisture content of ~53% (wb), distributed evenly in the central compartment of the dryer, considering a layer height of 0.20 m.

#### 2.1.2. Mechanical Dryer Model

**Figure 1:**
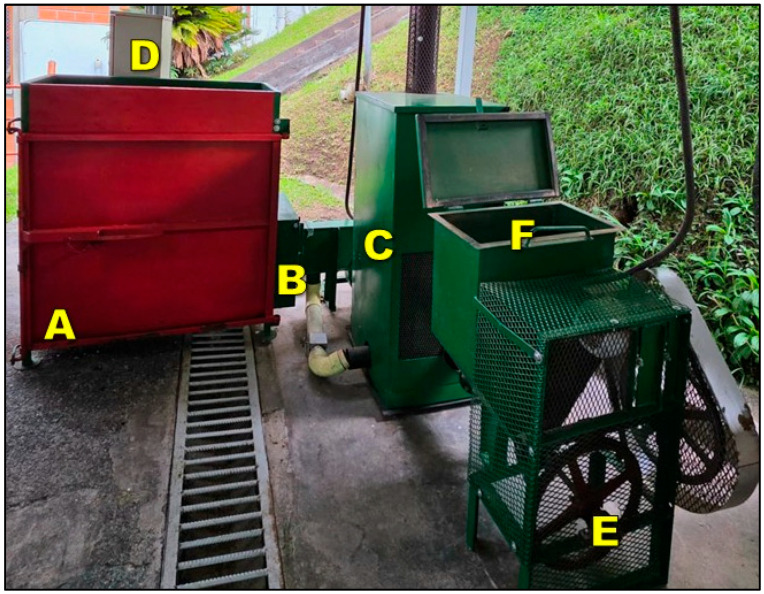
Experimental mechanical coffee dryer and main components. A: static-layer drying chamber with three levels; B: hot air inlet duct; C: coffee husk-fueled heat exchanger; D: Control panel; E: automatic biofuel feeding system; F: coffee husk deposition hopper.

The equipment evaluated is a mechanical dryer with a capacity of 125 kg of dry parchment coffee (dpc) per batch, featuring a rectangular carbon steel structure (Figure 1). The system consists of a static-layer drying chamber with three vertically stacked levels (Figure 1A), each with a perforated floor to allow vertical airflow. The grain transfer between levels is carried out by means of removable gates in the trays of the first and second compartments. The drying air, driven by an electric fan, enters the system through a hot air inlet duct after being heated in a coffee husk-fueled heat exchanger (Figure 1B,C). Each chamber has a volume of 0.1103 m^3^ and a cross-sectional area of 0.4798 m^2^, with a capacity for 75 kg of wet coffee (40 kg dpc) [35]. The plenum chamber at the bottom has a volume of 0.0408 m^3^. Air is supplied at 50 ± 2 °C, with a minimum flow rate of 0.1 m^3^ min^−1^ kg^−1^dpc [19]. The system incorporates a control panel equipped with a thermometer at the fan outlet, whose signal activates the automatic biofuel feeding mechanism, regulating the supply of coffee husk from the deposition hopper and ensuring thermal stability during the drying process (Figure 1D–F).

#### 2.1.3. Thermal Insulation Material

The selection of expanded polystyrene (EPS) as an insulating material is based on its thermal properties, physical stability, availability and low acquisition costs, qualities that make it a viable alternative to mitigate heat losses due to conduction and convection in mechanical coffee drying systems [31,32]. Table 1 presents the main physical and thermal characteristics of the EPS.

#### 2.1.4. Data Collection

**Figure 2:**
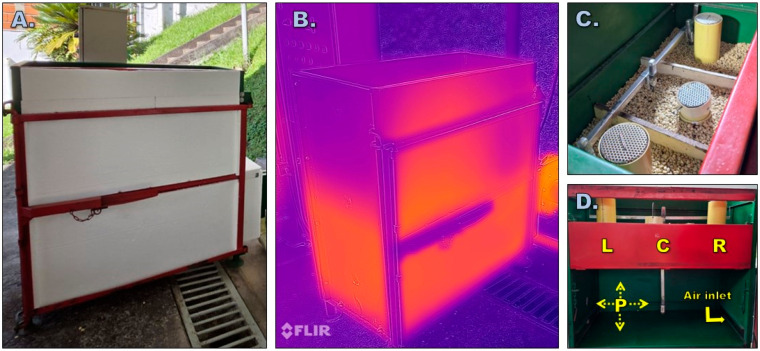
Evaluation process. (**A**) Drying chamber externally insulated with expanded polystyrene (EPS). (**B**) Measurement of the external surface temperature of the drying chamber using a thermal camera. (**C**) Internal configuration of the drying bed, showing the division of the static coffee layers and the location of grain temperature and moisture sensors. (**D**) Schematic internal view of the dryer indicating the position of the drying layers (left: L, center: C, right: R), the plenum chamber (P), and the direction of the air inlet.

The process was controlled until the hygroscopic equilibrium moisture of the coffee (10–12% wb) was reached, defined as the condition at which the grain neither gains nor loses moisture under the prevailing air conditions. Environmental variables, including temperature and relative humidity, were monitored both inside and outside the drying chamber using thermohygrometers (UT330B-IP67, UNI-T—China; accuracy ±1 °C and ±3%RH). In addition, the surface temperatures of the inner and outer walls were measured using a thermal camera (Lepton 3.5, FLIR, Wilsonville, OR, USA; accuracy ± 2 °C) (Figure 2B). Grain temperature and moisture were measured at different positions within the drying bed, as illustrated in Figure 2C,D.

Grain temperature was measured using a temperature sensor (LM35, Texas Instruments Inc., Dallas, TX, USA; accuracy ±1 °C) installed inside a thermally insulated vessel. Moisture content was determined using the Gravimet SM^®^ method (Figure 2C) and were indirectly verified with a capacitance moisture determiner (PM-450, Kett, Los Angeles, CA, USA; accuracy ±0.5% wb). Before each test, the initial moisture of the mass was obtained gravimetrically using the oven method with three samples of 10 g each (ISO 18134-3:2022 [41]). To evaluate the internal distribution of environmental variables, the drying bed was divided into three sectors (left: L; center: C; and right: R) (Figure 2C,D). Measurements were taken at hourly intervals, with the grain layer turned every 6 h. In addition, the total drying time as well as fuel and energy demand were recorded.

#### 2.1.5. Performance Parameters

##### Real System Efficiency

The real efficiency of the system ($\eta_{R}$, %) was determined using Equation (1) [15,19] where ${\dot{m}}_{a}$ is the air mass flow rate (995.7 kg h^−1^), calculated from the coffee mass in the dryer, the air flow rate, and the air density. The air flow rate was obtained from the ratio between volumetric flow and bed cross-sectional area, considering the pressure drop between two points at different heights within the grain layer, following Parra et al. [19]. $C_{pa}$ is the specific heat of air at constant pressure at 50 °C (1.005 kJ kg^−1^ K^−1^), ΔT is the air temperature difference (°C), ${\dot{m}}_{f}$ is the fuel consumption rate expressed as energy input (kJ h^−1^), and $h_{B}$ is the net calorific value (NCV) of the biofuel (kJ kg^−1^). Experimental values were substituted according to the test results.

(1) $$\eta_{R}=\frac{{\dot{m}}_{a}\;C_{pa}\;\Delta T}{{\dot{m}}_{f}\;h_{B}}$$

##### Drying Rate

The continuous recording of parameters such as grain moisture and drying time, made it possible to evaluate the process performance according to the drying rate ($DR,\;\%\;h^{-1})$, which quantifies the rate of moisture transfer from the coffee mass to the environment per unit of time [15,42,43], as shown in Equation (2).

(2) $$DR=\frac{M_{0}-M_{f}}{t}$$

where $M_{0}$ and $M_{f}$ refer to the initial and final moisture content of the sample (wb, %), respectively, and t is the drying time (h).

##### Real Fuel Consumption

Fuel consumption was determined by weighing a known initial mass of coffee husk (40 kg) and recording the mass of each controlled refill during the drying process. The total fuel consumption was calculated as the sum of the initial load and all refills. Fuel performance is then expressed as the ratio between the mass of dry parchment coffee produced (kg dpc) and the total biomass consumed during each drying cycle.

##### Electrical Energy Demand

Total electricity demand (*E*, kWh) was calculated according to Equation (3), considering the electrical power of the motor (*P*, kW) operating the fan and the automatic fuel supply system, in relation to the total drying time (h) [44].

(3) E=P t

#### 2.1.6. Coffee Bulk Parameters

##### Bulk Diffusion Coefficient

In the analysis of the drying process, the diffusion coefficient (*DC*) describes the transfer of moisture from the grains to the chamber environment by diffusion (m^2^ s^−1^). This parameter was calculated using Equation (4), according to Montoya et al. [45].

(4) $$DC=4.1582\times 10^{-8}e^{[\left(0.1346T_{g}+2.2055\right)M_{\left(db\right)}-\frac{1184}{T_{g}+273.15}}$$

where $T_{g}$ is the grain temperature (°C) and $M_{\left(db\right)}$ is the moisture content of the grain expressed in decimal notation and on a dry basis (db).

##### Bulk Specific Heat Capacity and Thermal Conductivity

The specific heat capacity ($C_{p}$, kJ kg^−1^ K^−1^) was obtained using Equation (5) developed by Duque-Dussán et al. [9] for the Cenicafé 1 variety. In this equation, $M_{\%\left(wb\right)}$ corresponds to the grain moisture content expressed as percentage on a wet basis. This property varies significantly with the moisture content of the material, from initial conditions ~53% (wb) to a final range of 10–12% (wb):

(5) $$C_{p}=5.40\;M_{\%\left(wb\right)}+1.178$$

Additionally, the bulk thermal conductivity (*K*, W m^−1^ K^−1^) was found using Equation (6), as proposed by the same reference [9], taking into account the variation in grain moisture content on a wet basis during the drying process for this variety.

(6) $$K=0.00241\;M_{\%\left(wb\right)}+0.0104$$

### 2.2. Experimental Design and Data Analysis

A completely randomized experimental design was used with two treatments: T1, insulated dryer using 0.02 m thick expanded polystyrene (EPS) sheet, and T2, control without insulation. For each treatment, the environmental variables of the chamber, drying time, fuel consumption, energy demand, drying rate, thermal efficiency of the system, and grain parameters associated with mass and energy transfer processes were calculated. Each treatment was replicated three times independently (*n =* 3), completing a total of six drying cycles.

For the variables of system efficiency, drying time, electricity demand, coffee husk consumption and yield, a one-way analysis of variance (*p* < 0.05) was used prior to compliance with the assumptions of normality (Shapiro–Wilk test) and homogeneity of variances (Levene test). When significant differences were detected, a multiple comparison test (Tukey HSD, *p* < 0.05) was applied to identify contrasts between means. For environmental variables, a nonparametric Kruskal–Wallis test was applied, followed by the Dunn–Bonferroni post hoc test (*p* < 0.05). Descriptive analyses were performed for the bulk parameters, the theoretical evaluation of the materials, and the economic analysis, considering average values and the behavior of the variables over time. Statistical processing was performed in R software (version 4.3.0) using stats and rxstatix packages, while graphical representation was performed in SigmaPlot (version 11.0).

### 2.3. Theoretical Model of Thermal Losses

#### 2.3.1. Heat Flow with Thermal Resistances in Series

The steady-state heat flow through the dryer walls (*Q*, W) was modeled by considering the system as a series of thermal resistances, where each layer contributes to the total thermal resistance [46]. These resistances include convection at the internal and external surfaces and conduction through the dryer wall materials and the thermal insulation, according to Equation (7), as proposed by Bergman et al. [47].

(7) $$Q=\frac{\Delta T}{\left[\left(\frac{1}{h_{1}\;A_{d}}\right)+\left(\frac{L_{S}}{k_{S\;}A_{d}}\right)+\left(\frac{L_{A}}{k_{A\;}A_{d}}\right)+\left(\frac{1}{h_{3}\;A_{d}}\right)\right]}$$

where ΔT is the temperature difference between the interior and exterior surfaces (°C), $h_{1}$ and $h_{3}$ are the internal and external convective heat transfer coefficients (W m^−2^ K^−1^), and $k_{S}$ and $k_{A}$ are the thermal conductivities of the wall material and insulation, respectively (W m^−1^ K^−1^); both parameters were obtained from literature for similar materials and operation conditions [32,48,49]. $L_{S}$ and $L_{A}$ are the corresponding material thicknesses (m), and $A_{d}$ is the evaluated heat transfer area (m^2^).

#### 2.3.2. System Thermal Efficiency and Biofuel Consumption

To determine both variables, the overall thermal efficiency of the system ($\eta_{MD},\;\%$) was defined, expressed as the equivalence between the useful energy used in coffee drying and the sum of all energy demands (Equation (8)) [42,43,50].

(8) $$\eta_{MD}=\frac{Q_{u}}{Q_{u}+Q_{a}+Q_{l}}$$

From this definition, fuel consumption $\left({\dot{m}}_{f}\right)$ was calculated as:

(9) $${\dot{m}}_{f}=\frac{Q_{u}+Q_{a}+Q_{l}}{\eta_{com}\;h_{B}}$$

where $Q_{u}$ corresponds to the energy used in grain heating and water evaporation during the drying time, whose value was calculated at 19,124 kJ h^−1^. $Q_{a}$ is defined as the energy needed to heat the air mass delivered by the heat exchanger (19,718 kJ h^−1^), while $Q_{l}$ represents heat losses by convection and conduction through the walls of the equipment, calculated using Equation (1) in kJ h^−1^. The theoretical combustion efficiency ($\eta_{com}$) was assumed as 50% and $h_{B}$ is the NCV of coffee husk (18,550 kJ kg^−1^) [51].

### 2.4. Economic Analysis

#### 2.4.1. Estimation of the Annual Operation Costs

The calculation of the annual operating costs ($A_{c}$) was carried out using Equation (10), given as the sum of three fundamental components [52,53]: fixed costs ($Co_{f}$), which include the depreciation of equipment and thermal insulation (assumed to 10 and 2 years, respectively); variable costs ($Co_{v}$), associated with fuel and electricity demand and maintenance costs ($Co_{m}$), estimated as 5% of fixed costs [54].

(10) $$A_{C}=Co_{f}+Co_{v}+Co_{m}$$

Thus, the useful life of the equipment was estimated at 10 years and 2 years for the insulating material; the dryer capacity is 125 kg dpc per cycle and 36 cycles per year were considered, considering its design for coffee growers with productions close to 4500 kg of dpc year^−1^. Coffee husk cost per kilogram was $0.05 USD, while the unit cost of energy was based on the average reported by the service provider for the first half of 2025: $0.20 USD kWh^−1^ [19,55].

#### 2.4.2. Payback Period and Return on Investment

To determine the recovery period (Pb), Equation (11) was considered, taking into account the initial cost of the investment ($I_{c}$) which includes the cost of the dryer and the insulated cover, together with the annual gross cash flow ($GF_{b}$) and operating costs ($O_{c}$). Fixed costs were excluded from operating costs in the payback analysis to avoid double counting of capital recovery [56,57].

(11) $$Pb=\frac{I_{c}}{GF_{b}-O_{c}}$$

(12) $$ROI_{annual}=\frac{GF_{b}-O_{c}}{I_{c}}$$

Similarly, the annual return on investment (ROI) was calculated using standard economic analysis methods based on Equation (12) [56,58]. These indicators make it possible to assess the economic viability of installing insulated covers on mechanical coffee drying systems.

## 3. Results

### 3.1. Experimental Evaluation

#### 3.1.1. Evaluation of Drying

**Figure 3:**
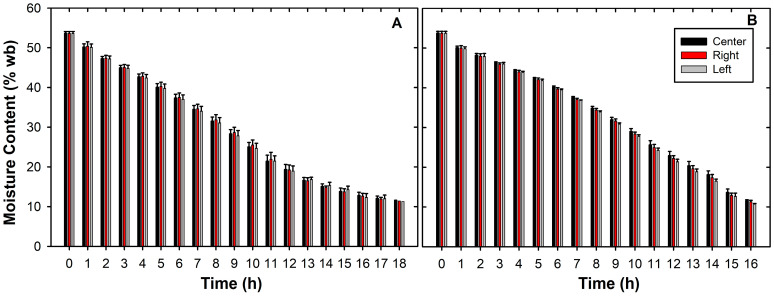
Drying rates. (**A**) Control. (**B**) EPS insulated. Each bar represents the average of the moisture content per sector in each hour of sampling (*n* = 3) ± Standard Error (SE).

The use of EPS increased the drying capacity of the equipment, decreasing the operating time from 17.0 h to 15.7 h on average, with final moisture contents of 11.58 ± 0.36% (wb) for control and 11.51 ± 0.35% (wb) with EPS (Figure 3). Both times are comparable to those reported for fixed-bed and static-layer mechanical coffee dryers operating at similar air temperatures (40–55 °C) [18,59]. It should be noted that, although there were no significant differences in drying times, this 7.82% reduction in time allows for an increase in processing capacity of approximately 8.5%, which is consistent with other studies on food drying, where there were lower losses and greater use of thermal energy within the system [25,60].

Regarding drying rates, the highest values were recorded for the EPS treatment (2.71 ± 0.12% h^−1^), compared with the control (2.49 ± 0.08% h^−1^). This increase confirms that the use of thermal insulation enhances the conservation of available thermal energy for water evaporation, thereby improving drying capacity, particularly in those sectors with higher airflow [18,61]. When analyzing the spatial distribution, the left side of the drying bed exhibited the highest drying rates in both treatments, with 2.49 ± 0.08% h^−1^ for the control and 2.74 ± 0.12% h^−1^ for the EPS treatment. This heterogeneity in drying dynamics is attributed to local differences in temperature, airflow, and air velocity associated with the dryer design [17,62,63].

**Figure 4:**
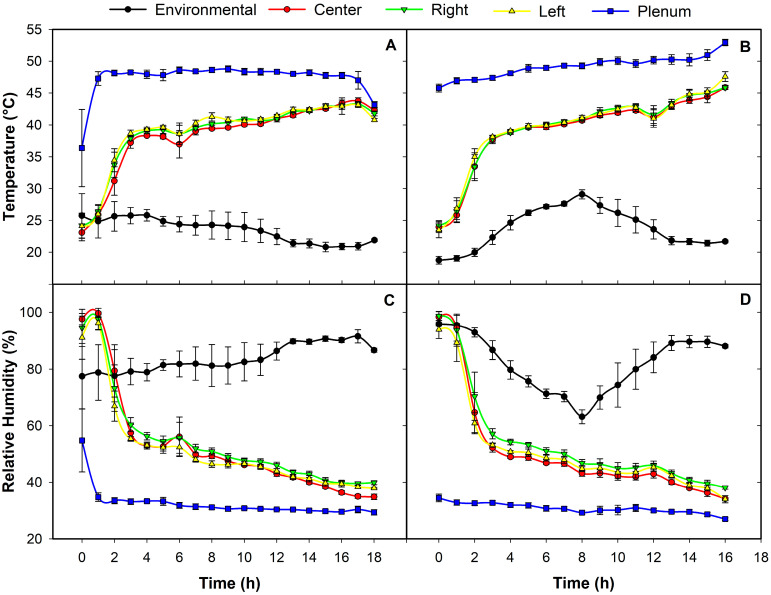
Temperature and relative humidity in the drying system and ambient air. (**A**) Temperature control. (**B**) Temperature with EPS insulation. (**C**) Control relative humidity. (**D**) Relative humidity with EPS insulation. Each point represents the mean (*n* = 3) ± SE.

As expected, the highest temperatures were recorded in the plenum chamber (*P*), with higher values under insulation conditions (49.1 °C vs. 47.5 °C without EPS). Once the drying air passed through the coffee bed, the left side (*L*) presented the highest temperatures in both treatments (39.4 °C with EPS and 39.0 °C control), while the lowest temperatures were obtained in the center (39.0 °C control and 38.8 °C with EPS) (Figure 4). Statistical analysis showed significant differences (*p* < 0.05) between treatments for the center (*C*), right (*R*) and plenum sectors, demonstrating that insulation effectively increases the temperature inside the drying chamber [64]. However, this distribution reflects that the flow of hot air toward the *L* sector may have been greater, while on the two sides near the air inlet there was greater saturation due to a lower temperature, which could compromise the uniformity of drying and explain the differences in moisture removal between the sectors [65,66,67].

In contrast, both treatments showed the highest relative humidity values on the right side (54.6% in the control and 54.2% in the EPS), while the lowest values were recorded on the left side in the control (52.0%) and in the center with the EPS (50.9%). It is important to note that relative humidity was measured in the air layer near the coffee mass, which reflects the local drying microclimate. In the distribution chamber, the average humidity decreased with insulation, from 32.5% in the control to 30.8%. On the other hand, the statistical analysis confirmed significant differences (*p* < 0.05) between treatments in the *C* and *P* sectors, indicating that the insulating material improves drying conditions by increasing the temperature and reducing the relative humidity [64]; in addition, low relative humidity values after the third hour support the operation of multiple-bed dryers, as the air still retains a high drying capacity [18,19]. Nevertheless, the observed variations in drying performance could indicate a distribution chamber design deficiency [15,68].

#### 3.1.2. Dryer Performance

The efficiency analysis showed reductions in fuel consumption, energy demand, and operating time of 13.9%, 9.5%, and 7.82%, respectively, when thermal insulation was used. This behavior indicates that EPS promotes better energy use, reducing heat losses to the environment and achieving faster drying, with lower energy demand and a 6.7% increase in system efficiency (Table 2). Similar findings have been reported in other drying systems with thermal collectors incorporating insulating materials, where higher thermal efficiencies are obtained due to the reduction in heat losses by conduction and convection [69,70,71].

**Figure 5:**
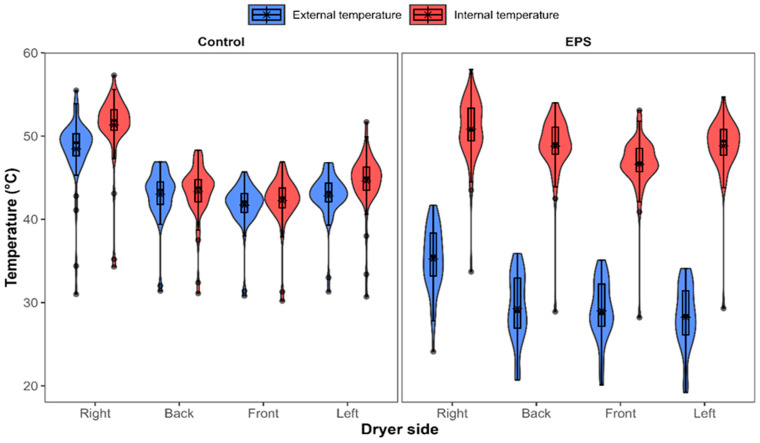
Internal and external surface temperatures of the drying chamber under control and EPS insulation conditions. Violin plots represent the distribution of temperature values at different sides of the dryer. Boxes indicate the interquartile range, and the asterisk represents the mean (*n* = 3).

During control tests, the internal and external walls reached average temperatures of 45.4 °C and 44.0 °C, respectively. With the use of EPS, internal temperatures were higher, with an average of 48.8 °C, while external temperatures dropped to 30.4 °C. This represents a 37.7% reduction in heat loss, with statistically significant differences between treatments (*p ≤* 0.0001). Higher internal temperatures and greater thermal uniformity were observed when the dryer was covered with thermal insulation; the highest temperatures were obtained on the right wall, corresponding to the hot air inlet zone, while the lowest temperatures were recorded at the front, where the chamber door was located (Figure 5).

The thermal energy losses observed in the control tests are attributed to the high thermal conductivity of the equipment manufacturing material (~50 W m^−1^ K^−1^) [49,72]. The addition of EPS decreased these losses due to its low thermal conductivity, which acted as a barrier to heat flow and allowed a higher and more stable internal temperature to be maintained [73]. This behavior is consistent with previous studies reporting improved internal thermal stability, lower thermal transmittance, and enhanced energy efficiency through the use of expanded polystyrene as a thermal insulation [64,74,75]; however, this property varies according to material density [76,77].

#### 3.1.3. Bulk Thermal Properties

**Figure 6:**
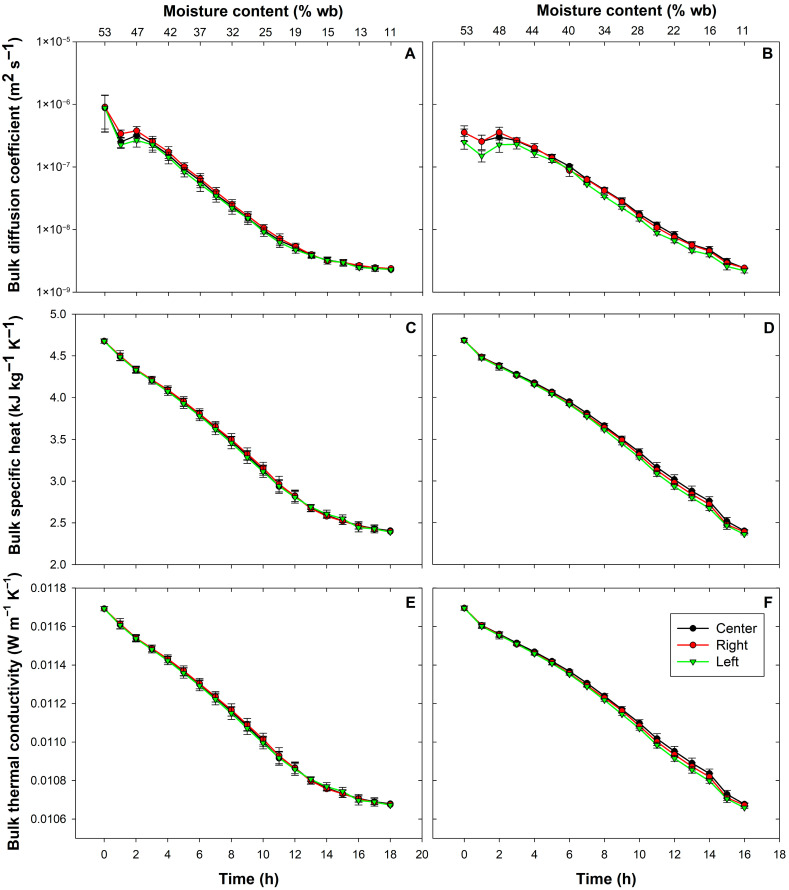
Time evolution of bulk thermal properties of parchment coffee during drying. Panels (**A**,**C**,**E**) correspond to the control treatment, while (**B**,**D**,**F**) represent the insulated system. Shown are the bulk diffusion coefficient (**A**,**B**), bulk specific heat (**C**,**D**), and bulk thermal conductivity (**E**,**F**) as a function of drying time and moisture content. Each point represents the mean (*n* = 3) ± SE.

The results in Figure 6 show that the temperature increase caused by the thermal insulation positively influenced the bulk diffusion coefficient of parchment coffee. This parameter decreased exponentially with drying time and moisture content, reflecting the reduced mobility of water as the material approached its hygroscopic equilibrium [9,18]. Slightly higher initial values were observed under insulation, attributed to the higher drying temperature that enhanced internal moisture transport. The estimated diffusion coefficients were within the ranges reported by other studies (8.88 × 10^−7^–2.32 × 10^−9^ m^2^·s^−1^ for the control and 3.19 × 10^−7^–2.35 × 10^−9^ m^2^·s^−1^ with insulation) [10,15,18]. After temperature stabilization around 50 °C, the insulated system exhibited a more uniform diffusion curve, particularly after 12 h, when the higher chamber temperature improved the drying dynamics. As the process approached equilibrium moisture, internal diffusion became the dominant mechanism, slowing moisture removal and increasing energy demand [78,79,80].

The bulk specific heat (Figure 6C,D) showed a decreasing trend with the reduction in moisture content, since water has a considerably higher heat capacity than the solid components of the grains. The differences between treatments became evident after 12 h of processing, thanks to the higher temperatures recorded with the use of EPS. This indicates that thermal insulation did not modify the composition or internal structure of the material but mainly influenced the drying environment conditions.

For its part, bulk thermal conductivity (Figure 6E,F) decreased almost linearly with time, in line with the progressive loss of moisture and increase in bed porosity. It is evident that the behavior of conductivity depends more on the moisture fraction than on temperature [17]. However, the influence of thermal insulation occurs at the end of the process due to the increase in chamber temperature, which increases energy retention within the grains and reduces the temperature gradient.

Considering that the determinant factors affecting moisture reduction are air flow, temperature, and air humidity [10], optimizing these variables, mainly temperature, allows the grains to increase the accumulation and transfer of thermal energy to its interior, reducing energy demands and processing time [9]. Overall, the results show that thermal insulation improves drying efficiency by increasing the temperature and reducing heat losses, which is reflected in higher diffusion coefficients during the early stages and more homogeneous coefficients at the end of the process. However, the intrinsic thermal parameters of parchment coffee (specific heat and thermal conductivity) remain essentially determined by the moisture content of the material and not by the external conditions of the system [9,17]. Therefore, thermal insulation—rather than altering surface convection—helps maintain more stable conditions inside the drying chamber, enhancing energy storage and heat transfer within the grain matrix.

### 3.2. Theoretical Model

To ensure homogeneity in the comparison, thicknesses of 0.02 m for all materials, a drying time of 16 h and a combustion efficiency of 50% were taken into account in the theoretical analyses, depending on the capacity of the equipment and the volatile matter of the fuel [51,59,81]. In addition to materials derived from the plastics industry such as EPS and polyethylene foam, insulation was selected from natural fibers such as glass wool/rock mineral wool, cork and bamboo fiber [32].

Theoretical analyses, based on steady-state heat transfer equations (Equations (7)–(9)), indicate that the implementation of thermal insulation allows a substantial reduction in energy losses compared to the non-insulated condition (1011.96 kJ h^−1^); however, the magnitude of this reduction varied with the type of insulation (Table 3) [31]. Expanded polystyrene was the material with the lowest heat transfer (355.58 kJ h^−1^), reaching a thermal efficiency of the system of 48.79%. Similar values were observed for polyethylene foam (391.89 kJ h^−1^; 48.74%) and glass wool/rock mineral wool (411.81 kJ h^−1^; 48.72%). These results show that the low thermal conductivity of the materials is the main factor responsible for their greater ability to mitigate heat losses [82,83].

Although materials such as cork and bamboo fiber showed higher losses (475.06 and 739.10 kJ h^−1^, respectively) compared to synthetic polymers, it has been shown that these types of naturally occurring insulators, such as cork and lignocellulosic fibers, although they have a higher thermal conductivity, offer advantages in terms of biodegradability, low carbon footprint and valorization of local resources [84,85].

In terms of overall efficiency, similar values were observed between the different materials and the non-insulated condition (Table 3). This shows that the influence of insulation on the overall efficiency of the process is not as marked as on heat losses, and that this depends largely on operational and design factors of the equipment [16,17,42]. Likewise, fuel consumption remained constant between all scenarios (~4.23–4.30 kg h^−1^), due to the fact that the flow rate did not change. As a result, the energy demand for air heating remained fixed [19,86]. Considering that airflow has the greatest impact on energy demand, drying time and efficiency [16,87,88], in future evaluations, modifications in the flow rate could be considered based on the energy storage capacity in the thermally insulated drying chamber.

When the acquisition cost of each material was compared, a critical difference in selection was identified. On the other hand, expanded polystyrene not only offers the best thermal performance, but also has the lowest cost (3.25 USD) compared to the other alternatives, considering local quotes. In this sense, although the thermal performance of the different materials is comparable, the cost–benefit ratio positions EPS as the most advantageous option for practical applications in rural environments, as it guarantees a significant reduction in heat loss with the lowest economic investment [83,89].

### 3.3. Economic Analysis

The economic analysis summarized in Table 4 compared the feasibility of a mechanical coffee drying system without thermal insulation (control) and with the use of EPS, considering an annual production of 4500 kg of dpc, taking into account that the dryer is designed for a coffee grower who owns between one and three hectares, with a peak productive day of 600 kg of cherry coffee [19,59].

The investment cost of purchasing the equipment was estimated at USD 2000, and the purchase and installation of the insulation material cost around USD 13, slightly increasing the initial investment for EPS treatment. Despite this increase, the improvement in operational performance resulted in economic benefits and greater annual drying capacity, in agreement with trends reported for energy-optimized coffee drying systems.

The addition of EPS insulation reduced drying time from 648 to 576 h for a volume of 4500 kg of dpc, resulting in an 11.11% increase in theoretical annual production capacity, from 60,833 kg to 68,437 kg of dpc when using 100% of the equipment’s capacity (125 kg per batch). Similar increases in throughput associated with thermal or energy-efficiency improvements have been reported in recent coffee drying studies, where reductions in processing time directly translated into higher annual production and lower unit drying costs [15,56]. This increased productivity, coupled with a reduction in annual operating costs from USD 723 to USD 635, increased annual profit by USD 82 in cost savings and 12.5% in increased earnings due to greater equipment utilization.

The reduced drying time and increased operational capability of the insulated system offset the marginally higher initial cost [90,91]. As for the Payback Period, it was estimated that the coffee grower will only allocate 20% of their income from the sale of 125 kg of dpc per batch, that is, $125 for the payment of drying equipment. This was reflected in a payback period of 0.45 years, very similar to that of the control system (0.44 years), confirming that the additional cost of insulation is quickly amortized by operational efficiencies [53,58,92]. Likewise, the return on investment (ROI) remained competitive, with values of 10.89 and 10.86 for the control and EPS systems, respectively, supporting the economic viability of the use of expanded polystyrene as an insulating material.

## 4. Conclusions

This study demonstrated, through theoretical and experimental analyses, that incorporating thermal insulation improves the thermal performance of a mechanical coffee dryer by reducing heat losses and enhancing thermal stability. Expanded polystyrene (EPS) exhibited the best performance due to its low thermal conductivity, while natural lignocellulosic materials remain promising sustainable alternatives with potential for further development.

Experimental results showed that EPS insulation increased drying efficiency by 6.73%, reduced drying time by 7.82%, and decreased fuel consumption by 13.90%. These improvements were associated with higher internal chamber temperatures and lower heat losses, confirming EPS as an effective thermal barrier that enhances process efficiency and reduces energy demand in postharvest coffee drying.

Improved drying conditions were reflected in the thermophysical behavior of the coffee bed, as indicated by changes in bulk specific heat capacity and thermal conductivity, describing the integrated response of the coffee–air matrix. Although a moderated moisture diffusion coefficient was observed, this behavior favored more uniform drying by reducing internal temperature gradients, limiting mechanical stress, and preserving bean integrity.

The novelty of this work lies in the integrated evaluation of thermal insulation effects on drying dynamics, thermophysical behavior, and economic performance in mechanical coffee drying, a field with limited experimental evidence. From an economic and societal perspective, the additional investment required for insulation was rapidly offset by increased processing capacity and reduced operating costs, demonstrating that EPS insulation is a low-cost, easily adoptable strategy to improve energy efficiency and strengthen the sustainability of small- and medium-scale coffee producers.

## Figures and Tables

**Table 1:** Thermal properties of Expanded Polystyrene (EPS).

Property	Typical Value	Unit	Reference
Density	10–30	kg m^−3^	[36]
Thermal Conductivity	0.030–0.040	W m^−1^ K^−1^	[37]
Thermal resistance	−50–80	°C	[38]
Fire Classification	E	-	[39]
Moisture absorption	1–5	% vol.	[40]

**Table 2:** Comparison of system efficiency, drying time, electrical demand, coffee husk consumption, and drying performance under control and EPS insulation treatments. CV: Coefficient of Variation, DF: Degrees of Freedom; F: Fisher Coefficient; SL: Significance Level; ns: non-significant.

Variable	Treatment	Mean	CV	DF	F	*p*-Value	SL
System Efficiency (%)	Control	43.23	5.12%	1.4	3.39	0.1390	ns
	EPS	46.14	6.40%				
Drying Time (h)	Control	17.00	5.88%	1.4	4.000	0.1160	ns
	EPS	15.67	3.69%				
Electricity Demand (kWh)	Control	14.31	5.68%	1.4	6.249	0.0668	ns
	EPS	12.95	4.74%				
Coffee husk Consumption (kg)	Control	52.07	7.86%	1.4	4.791	0.0923	ns
	EPS	44.83	7.05%				
Performance (kg husk kg^−1^ dpc)	Control	1.49	9.16%	1.4	3.991	0.1160	ns
	EPS	1.27	8.06%				

**Table 3:** Comparative thermal performance and cost of insulation materials. K, thermal conductivity of the insulation material; Q, steady-state heat flow through the dryer walls; $\eta_{MD}$, mechanical drying efficiency; ${\dot{m}}_{f}$, fuel consumption rate. See the nomenclature section for full definitions.

Material	K (W m^−1^ K^−1^)	Q (W)	$\eta_{MD}$ (%)	${\dot{m}}_{f}$ (kg h^−1^)	Cost (USD m^−2^)
Expanded polystyrene foam	0.030	98.77	48.79	4.23	3.25
Polyethylene foam	0.035	108.86	48.74	4.23	4.25
Glass/rock mineral wool	0.038	114.39	48.72	4.23	15
Cork	0.049	131.96	48.64	4.24	100
Bamboo fiber	0.150	205.30	48.32	4.27	40 *
Non-insulated dryer	-	281.10	47.98	4.30	-

* Considering the wide availability of materials such as Bamboo (*Guadua angustifolia*) on farms, it can be manufactured directly on-site.

**Table 4:** Economic analysis and performance indicators of mechanical dryer with insulation (EPS) and control.

Parameter	Control	EPS Insulation
$T_{c}$ (USD)	2000	2013
$Co_{f}$ (USD)	200	206
$Co_{v}$ (USD)	703	614
$Co_{m}$ (USD)	20	21
$A_{c}$ (USD)	923	841
$O_{c}$ (USD)	723	635
Drying time (h) (Dq = 4500 kg)	648	576
Drying cost (USD kg^−1^dpc)	0.205	0.187
Theoretical annual capacity (kg)	60,833	68,437
Annual profit (USD) (Dq—Ac)	21,577	21,659
Payback period (years)	0.44	0.45
Calculated ROI	10.89	10.86

## Data Availability

The data presented in this study are available on request from the corresponding author due to restrictions related to privacy and third-party ownership. The dataset contains information generated with Colombian coffee growers and remains their property; therefore, it cannot be deposited in a public repository without their authorization.

## Abbreviations

The following abbreviations are used in this manuscript:

Moisture Content and Temperature		Energy Demand and Thermal Efficiency	
*DR*	Drying rate (%)	*E*	Electricity demand (kW h^−1^)
M	Moisture content (%)	*P*	Electric power motor (kW)
$M_{0}$	Initial moisture content (%)	*t*	Total drying time (h)
$M_{f}$	Final moisture content (%)	$Q_{u}$	Useful drying energy (kJ h^−1^)
*T*	Temperature (°C)	$Q_{a}$	Air heating energy (kJ h^−1^)
ΔT	Air temperature difference (K)	$Q_{l}$	Heat loss energy (kJ h^−1^)
$T_{g}$	Grains temperature (°C)	$h_{B}$	Fuel specific energy (kJ kg^−1^)
		${\dot{m}}_{a}$	Air mass flow rate (kg h^−1^)
Heat and Mass Transfer		${\dot{m}}_{f}$	Biofuel consumption (kg h^−1^)
*DC*	Bulk diffusion coefficient (m^2^ s^−1^)	$\eta_{com}$	Combustion efficiency (%)
$C_{p\;air}$	Specific heat capacity of air (kJ kg^−1^ K^−1^)	$\eta_{MD}$	Theoretical system thermal efficiency (%)
$C_{p}$	Bulk specific heat transfer (kJ kg^−1^K^−1^)	$\eta_{R}$	Experimental efficiency (%)
$G_{a}$	Mass transfer per unit time (kg s^−1^ m^−2^)		
*K*	Bulk thermal conductivity (W m^−1^ K^−1^)	Economic Analysis	
$r_{0}$	Longest dimension of wet grain (m)	Ac	Annual costs (USD)
Q	Heat flow in thermal resistances (W)	$Co_{f}$	Fixed costs (USD)
$h_{1,3}$	Heat transfer coefficient (W m^−2^ K^−1^)	$Co_{v}$	Variable costs (USD)
$k_{S,A}$	Thermal conductivity (W m^−2^ K^−1^)	$Co_{m}$	Maintenance costs (USD)
$L_{S,A}$	Material thickness (m)	*Pb*	Payback period (years)
*A_d_*	Evaluated area (m^2^)	*ROI*	Annual Return on Investment
		*Ic*	Initial costs (USD)
		$GF_{b}$	Gross Cash Flow (USD)
		$O_{c}$	Operation cost (USD)
